# Progression to End-Stage Renal Disease Due to IgG4-Related Nephritis Refractory to Rituximab

**DOI:** 10.7759/cureus.36327

**Published:** 2023-03-18

**Authors:** Tien Nguyen, Sergey Brodsky, Natallia Maroz

**Affiliations:** 1 Internal Medicine, Kettering Health Main Campus, Dayton, USA; 2 Pathology, The Ohio State University Wexner Medical Center, Columbus, USA; 3 Internal Medicine, Kettering Health Network, Kettering, USA; 4 Nephrology, Wright State University, Dayton, USA

**Keywords:** igg4-rd, storiform fibrosis, tubulointerstitial nephritis, rituximab therapy, igg4-related kidney disease

## Abstract

An 81-year-old woman was referred to nephrology for a follow-up on progressive chronic kidney disease. She has a past medical history of hypertension, T2DM, breast cancer, and secondary hyperparathyroidism related to renal disease. A renal biopsy showed patchy interstitial fibrosis and tubular atrophy with an increased number of IgG4-positive plasma cells. A diagnosis of IgG4-related kidney disease was made based on clinical presentation and pathology. The patient ultimately required the initiation of hemodialysis, despite the administration of steroids and rituximab.

## Introduction

Immunoglobulin G4-related disease (IgG4-RD) is a rare systemic inflammatory disorder affecting multiple organs and tissues [1]. It is characterized by a typical histopathological pattern of abundant infiltration of IgG4+ plasma cells and interstitial storiform fibrosis in the involved organs, which most frequently involve the pancreas, retroperitoneum, lymph nodes, and salivary glands [1]. IgG4-RD may involve the kidney directly (IgG4-related kidney disease, IgG4-RKD), which often presents as tubulointerstitial nephritis (TIN) [2]. Approximately 75% of patients presenting with IgG4-RKD have disease manifestations in other organs in addition to the kidneys [2]. Recognition of IgG4 renal disease remains a challenge due to its varied clinical course and the absence of clearly defined diagnostic criteria. We present a case of IgG4-related kidney disease that was treated with steroids and rituximab and ultimately required hemodialysis.

## Case presentation

An 81-year-old female with a past medical history of hypertension, secondary hyperparathyroidism related to renal disease, type 2 diabetes mellitus, breast cancer (not on chemotherapy), and osteoporosis presented to the hospital with worsening serum creatinine. She had an unintentional weight loss of 50 lb in less than a year and was on chronic proton pump inhibitor therapy for Barrett’s esophagus. She had no history of smoking, habitual drinking, or renal disease. The vital signs and physical examination were unremarkable. Medications at the time included amlodipine 10 mg daily, atorvastatin 10 mg daily, carvedilol 12.5 mg twice daily, Coenzyme Q10 400 mg daily, vitamin B12 100 mcg daily, fexofenadine 60 mg daily, gabapentin 500 mg daily, levothyroxine 88 mcg daily, mirtazapine 15mg daily, omeprazole 20 mg daily, and torsemide 40 mg daily.

**Figure 1:**
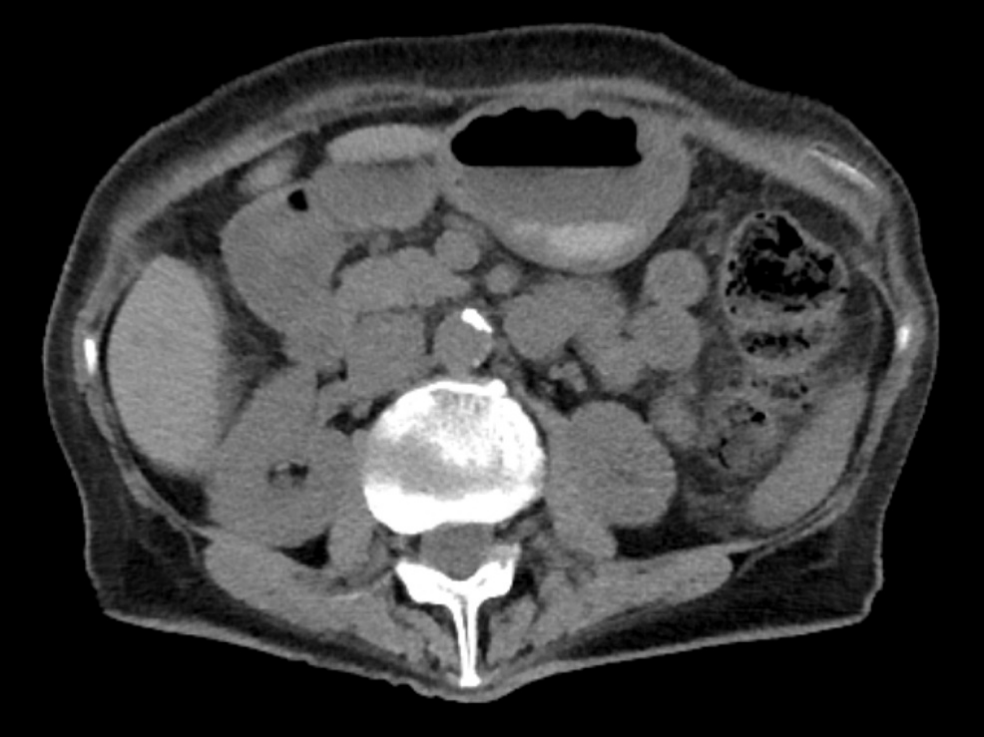
CT abdomen/pelvis demonstrates left renal atrophy with no hydronephrosis or nephrolithiasis.

Laboratory analysis revealed an increase in creatinine from 1.04 to 2.82 in eight months. Other significant laboratory values included a urine protein to creatinine ratio of 0.6, anemia with hemoglobin 8.2 g/dl, and a blood urea nitrogen (BUN) level of 108 mg/dL. Hemoglobin A1c is 8.3%. Urinalysis demonstrated pH 6, specific gravity 1.007, trace protein, and 0-5 white blood cells; no blood, red blood cells, or crystals were present. Serum and urine protein electrophoresis (SPEP and UPEP) were negative for monoclonal proteins. Testing was negative for ANA, ANCA, RNP, scleroderma, SS-A, SS-B, hepatitis markers, and immunofixation. Antihistone and anti-double-stranded DNA antibodies were positive. Complement levels were within normal limits: C3 93 mg/dL and C4 19 mg/dL. Computed tomography (CT) without contrast showed an atrophic left kidney (Figure 1). A percutaneous renal needle biopsy of the right kidney showed patchy interstitial fibrosis and tubular atrophy with inflammatory cell infiltrates and an increased number of plasma cells. There were clusters with more than 10 IgG4-positive plasma cells per high-power field detected by immunoperoxidase staining (Figure 1). No immune complex deposition or monoclonal immunoglobulin deposition was detected by immunofluorescence and electron microscopy. Serum IgG4 levels were elevated at 384 mg/dL. The diagnosis of IgG4-RKD was reached based on the above findings. She was treated with rituximab in lieu of steroids due to her uncontrolled diabetes and known osteoporosis. Unfortunately, despite receiving two doses of 1gm of intravenous rituximab two weeks apart, her creatinine worsened to 4.2 mg/dL. Her kidney function was monitored in close correlation with IgG4 levels to guide therapy.

Five months after her initial presentation to the nephrology clinic, she was referred to the emergency department due to abnormal outpatient labs, which were as follows: Hgb 7.7 g/dL, BUN 76 mg/dL, Cr 7.2 mg/dL, CO2 13, anion gap 16, Cl 111 mg/dL, Ca 8.3 mg/dL. She did not have any uremic symptoms. Additional laboratory serological findings include C3 of 96 mg/dL, C431 mg/dL, ESR of 14 mm/hr, microalbumin/creatinine ratio of 50.7 mg/g, urine protein of 34 mg, urine creatinine of 25.72 mg/dL, and urine protein/creatinine ratio of 1.3 mg/dL. IgG4 level of 139mg/dL. Renal ultrasound revealed atrophy of the left kidney along with a new lobulated appearance of the right kidney when compared to imaging from eight months prior. She was treated with intravenous methylprednisolone, sodium bicarbonate infusions, blood transfusions, and electrolyte replenishment. Her creatinine level minimally improved to 6.3 mg/dL. Her medications at the time of discharge included sodium bicarbonate (1300 mg) three times daily, prednisone (50 mg) daily, calcium acetate (667 mg) three times daily, and potassium chloride (20 mEq) daily. 

Unfortunately, a few weeks before her outpatient follow-up appointment, she was readmitted with symptoms of weakness, falls, and lower extremity swelling. Her laboratory markers again demonstrated severe renal impairment, including a BUN of 108 mg/dL and a Cr of 6.6 mg/dL. Due to the progressing renal insufficiency associated with uremic symptoms, hemodialysis was initiated. Upon discharge, sodium bicarbonate was discontinued, and she was given a prednisone taper. Hemodialysis was continued as an outpatient, and no signs of renal recovery were observed for four months. 

Since her initial presentation 10 months ago, her appetite has improved, and she has been able to gain weight and return to independent living. The patient has achieved clinical stability without patient medical management and without requiring further hospitalizations.

## Discussion

Since IgG4-related disease can involve multiple organs, it can be mischaracterized for a variety of conditions, including malignancy, infection, SLE, Sjogren’s syndrome, or vasculitis (ANCA-associated vasculitis). In this case, no features were present in the history to indicate a drug-induced or infective cause of tubulointerstitial nephritis. The clinical features, serology, and histopathologic findings were not suggestive of SLE. Similarly, the positive antibodies seen in immune-mediated conditions such as Sjogren’s or ANCA-associated vasculitis were absent. Other histological findings usually present on a renal biopsy, such as necrotizing or crescentic glomerulonephritis or granulomas, were not observed.

The IgG4-related disease is a relatively new clinical entity first described in 2003 and explains the phenomenon of unrelated entities such as type I autoimmune pancreatitis (AIP), retroperitoneal fibrosis, Riedel’s thyroiditis, and sclerosing cholangitis occurring simultaneously in a multitude of patients and sharing common histological findings [3]. The global incidence and prevalence of IgG4-RD are largely underestimated, and typically middle-aged and older males are affected. There are clear associations between environmental and genetic risk factors and IgG4-RD that remain to be discovered. Early detection of IgG4-RKD provides the best chance for preserving renal function.

Although IgG4-RD has been brought to increasing attention by clinicians worldwide, the disease is still misdiagnosed as neoplastic, inflammatory, and infectious conditions. Serological findings are largely non-specific, including inflammatory markers such as ESR and CRP, which can be mildly elevated [1]. Some have low titers of antinuclear antibodies and/or a positive rheumatoid factor [4]. Serum IgG4 elevation occurs in 55%-97% of patients and can correlate with the number of organs involved [4]. However, it has poor diagnostic utility because it can occur in malignancies, infections, and autoimmune conditions. Disease-specific autoantibodies such as ANCA, SSA/Ro, or SSB/La, dsDNA, RNP, and Sm are not observed in IgG4-RD. Radiological findings can be non-specific in most affected organs. Because of the deficiencies of serological and radiological findings, histological examination is the most reliable tool for a definitive diagnosis. The 2019 ACR/EULAR classification criteria for IgG4-RD provide useful guidelines for disease recognition [5]. Novel diagnostic biomarkers such as the ratio of serum IgG4 to total IgG, quantitative PCR of the IgG4:IgG RNA ratio, and multicolor flow cytometry have been proposed and are awaiting validation in large prospective multicenter studies.

Based on the 2019 American College of Rheumatology/European League Against Rheumatism classification criteria for IgG4-related disease, our patient scored 21. The criteria are based on the absence of exclusion criteria, histopathologic findings, immunostaining, serum IgG4 concentration, and organ involvement, with a cutoff score of 20 [5]. Our patient met entry criteria with clinical and radiologic involvement of a typical organ and did not meet exclusion criteria for other diseases. Histopathology revealed a dense lymphocytic infiltrate with fibrosis, and the serum IgG4 concentration was four times the upper limit of normal. Finally, imaging of the kidneys showed evidence of renal pelvis thickening.

Kawano et al. proposed diagnostic criteria for IgG4-RKD in 2011, which include organ dysfunction/damage, elevated total IgG, radiological findings such as multiple hypodensities, hypovascular masses, or renal enlargement, histopathological findings of IgG4-positive plasma cells, and storiform fibrosis [6]. Our patient met the proposed criteria, with clinical features and serology ruling out other disorders such as vasculitis or lupus. The presence of storiform fibrosis, a key finding in cases of IgG4-RKD, further strengthens our diagnosis of IgG4 disease. Compared to IgG-RD patients, IgG4-RKD patients more frequently show hypocomplementemia and elevated serum IgG4 levels [7]. Our patient had low, normal complement levels and elevated IgG4 levels.

The most common feature of renal involvement in IgG4-RD is tubulointerstitial nephritis with an abundance of IgG4+ plasma cells and storiform fibrosis located in the interstitium [8]. However, increased numbers of IgG4-positive plasma cells may be a nonspecific finding for IgG4-related TIN since they are also noted in diseases such as anti-neutrophil cytoplasmic antibody (ANCA)-associated glomerulonephritis, lupus nephritis, membranous glomerulonephritis, and diabetic nephropathy. CT imaging, usually without contrast for patients with impaired renal function, shows a diffuse enlargement of the kidney and multiple low-density lesions [8]. Renal involvement may also include a wide range of manifestations, such as membranous glomerulonephropathy, pyelitis, and hydronephrosis due to retroperitoneal fibrosis. Most patients with IgG4-RKD have IgG4-related extrarenal lesions in organs such as the salivary glands, lacrimal glands, pancreas, and lymph nodes [9,10]. In our case, our patient surprisingly did not have evidence of extra-renal manifestations of IgG4-RD. Thus, her IgG4-RD was restricted to the kidneys, as the patient had no other organ dysfunction or dermatologic findings to suggest otherwise. In a study by Kawano et al., out of 41 cases of IgG4-RKD, only two cases exhibited IgG4-RD limited to the kidney [6]. It was also reported that IgG4-RD frequently develops in three to four organs simultaneously [6]. 

There are seven case reports in which IgG4+ disease was localized solely in the kidneys, listed in Table 1. A search was conducted using PubMed with the terms “IgG4,” “renal,” “kidney,” “diagnosis,” and “treatment.” We excluded all case reports with extra-renal manifestations of IgG4. The studies were published between 2010 and 2019. The renal findings included predominantly tubulointerstitial nephritis and membranous glomerulonephritis (MGN). Based on the number of available studies and our search, which revealed nine patients documented in the literature, IgG4 disease limited to the kidneys is uncommon.

**Table 1:** Summary of IgG4 renal disease case reports and response to treatment. MGN: membranous glomerulonephritis, TIN: tubulointerstitial nephritis, MN: membranous nephropathy

Study	IgG4 Renal Manifestation	Number of patients	Treatment	Duration	Outcome
Anvar et al., 2021	MGN	1	prednisone	6 months	Proteinuria partial response, decrease in Cr (6.6→2.5). (Normal Cr 0.7-1.3 mg/dL)
Matthai et al., 2017	TIN	1	steroids	1 year	Minimal improvement Cr 5.5→3.68 (Normal Cr 0.7-1.3 mg/dL)
Ono et al., 2017	TIN	1	50 mg oral prednisolone	1 year	Decrease in Cr and IgG4 Cr 12.36→3.5 (Normal Cr 0.7-1.3 mg/dL)
Quattrocchio et al., 2018	TIN	3	Prednisone, cyclophosphamide plus 4 weekly Rituximab administrations	12 months	eGFR increased
Saeki et al., 2010	TIN+MN	1	steroids	4 weeks	Cr 1.48→1.39 (Normal Cr 0.7-1.3 mg/dL)
Zhang N et al., 2019	Nephrotic syndrome IgG4-related interstitial nephritis	1	Prednisone and cyclophosphamide	2 months	Renal function improved Cr 96 mmol/L→77→340→89
Zhang W et al., 2018	combined IgG4 membranous nephropathy and TIN	1	prednisone	24 months	proteinuria and hematuria both resolved Cr 2.5→1.63 (Normal Cr 0.7-1.3 mg/dL)

Glucocorticoids are the first line of therapy for both IgG4-RD and IgG4-RKD [11,12]. The Saeki et al. case demonstrated a rapid improvement in creatinine levels following treatment with steroids in over 90% of patients [13]. However, disease relapse after discontinuing treatment with glucocorticoids is common, and long-term adverse effects have prompted a search for more effective options [12]. Rituximab is frequently used as second-line therapy, especially in refractory cases [14-17]. Khosroshahi et al. demonstrated that treatment with rituximab promptly led to clinical and serologic improvement in patients with refractory IgG4-RSD [18]. Rituximab appeared to be an effective treatment even without glucocorticoid therapy in a trial by Carruthers et al. that compared rituximab treatment to rituximab with concomitant glucocorticoid therapy [19]. Thus, serial treatments with rituximab may lead to progressive declines in serum IgG4 concentrations and better disease control. 

According to Table 1, all of the patients with IgG4 disease restricted to the kidneys were treated with steroids. Two patients were given cyclophosphamide in addition to prednisone [12,14]. Three patients were treated with prednisone, cyclophosphamide, and rituximab weekly for one month [12]. The duration of treatment ranged from 4 weeks to 24 months. In all cases, there was an improvement in renal function as measured by a decrease in serum creatinine or eGFR. In regards to our patient, she was initially treated with two doses of rituximab in lieu of steroids due to her history of diabetes. Due to worsening renal function, she was then given steroids for a month but ultimately required initiation of long-term dialysis for end-stage kidney disease. 

## Conclusions

The multiorgan involvement of IgG4-RD makes the diagnosis a challenge. Clinical presentations may vary due to the manifestation of the same condition in different organ systems. It is important for physicians of all specialties in which IgG4-RD may be present to not only be aware of the diagnostic criteria but also to be knowledgeable about the treatment to prevent progressive or irreversible disease, especially regarding renal dysfunction. Any delay in treatment may increase the risk of kidney failure. Most recently, the first classification criteria for IgG4-RD were developed by the American College of Rheumatologists and the European League Against Rheumatism, which will facilitate the identification of more patients with this disease. Treatment for IgG4 disease restricted to the kidneys, according to the literature, has included steroids as first-line therapy, rituximab for refractory disease, and hemodialysis in severe cases. This is the first case observing the progression to end-stage kidney disease from IgG4-RD, per the author’s best knowledge. Given our patient’s variable responses to the provided treatments, we wanted to share our experience with clinicians managing similar cases. 
